# Tuberous Sclerosis Associated With Aortic Stenosis and Endocarditis: A Case Report

**DOI:** 10.7759/cureus.38480

**Published:** 2023-05-03

**Authors:** Laura Duque González, David Ocampo Moreno, Alejandro Echavarria Cross, Sergio Franco Sierra, Tomas Escobar Gil

**Affiliations:** 1 Cardiology, Hospital San Vicente Fundación Rionegro, Rionegro, COL; 2 Cardiology, Universidad CES, Medellin, COL; 3 Internal Medicine, Universidad CES, Medellín, COL; 4 Cardiothoracic Surgery, Hospital San Vicente Fundación Rionegro, Rionegro, COL; 5 Internal Medicine, University of New Mexico School of Medicine, Albuquerque, USA

**Keywords:** infectious endocarditis, subvalvular membrane, heart tumor, aortic stenosis, angiomyolipoma, tuberous sclerosis

## Abstract

Tuberous sclerosis (TS) is a multisystem neurocutaneous disorder with an autosomal dominant pattern of inheritance. It is characterized by hamartomas that damage the skin, kidneys, lungs, heart, and central nervous system, among other organs. Rhabdomyomas, benign tumors of aberrant myocytes, are common in affected patients at birth. Depending on their size and location, these lesions might create valvopathies, which can cause heart failure or malignant arrhythmias, or they can cause obstruction of the outlet or inlet tract. Before making the diagnosis, a long time-even years-often passes. Early diagnosis can help prevent permanent irreversible complications. Differential diagnoses may include neurofibromatosis type 1, Sturge-Weber syndrome, and von Hippel-Lindau disease, among others. Diagnostic aids, such as MRI, CT scans, and genetic testing, can be useful in confirming a diagnosis of TS. Histological findings may include the presence of hamartomas, which are benign tumors composed of abnormal cells. Treatment for TS is mainly supportive and may involve medications to manage symptoms, and surgery to remove tumors. We present the case of a 23-year-old woman with TS who was admitted with macroscopic hematuria and fever, with further workup revealing tumor-like cardiac lesions associated with infective endocarditis.

## Introduction

Tuberous sclerosis (TS) is a multisystem neurocutaneous disorder with an autosomal dominant pattern of inheritance. It occurs by deletion, rearrangement, and inactivation of tumor suppressor genes (*TSC1 *or *TSC2*). It is characterized by hamartomas that affect multiple organs, including the central nervous system (CNS), skin, kidneys, lungs, and heart. This condition affects 1/6,000-1/10,000 live births, without gender or race preferences [1-3]. Approximately 66%-75% of affected patients have rhabdomyomas (benign tumors of abnormal myocytes) at birth. Although these lesions are usually asymptomatic, they depend on the size and location if they produce obstruction of the outlet/inlet tract or valvopathies, leading to heart failure or malignant arrhythmias [1,2,4]. According to the literature, these masses can be detected as early as 22 weeks of gestation and typically disappear entirely during the early years of life [1-3,5]. In a cohort study from Northern Ireland, almost 20% of patients were diagnosed later than 15 years old [6]. In United States cohorts, 12%-59% of cases were diagnosed in patients older than 21, and 7% of cases were diagnosed between ages 11 and 20 [7,8]. There is limited data on the prevalence in low-income countries, but it is believed to occur at similar rates worldwide, and hits occurrence does not appear to be influenced by socioeconomic status or geographic location. The delay in diagnosis was an average of 21.5 years; of these, 66% met the diagnostic criteria in childhood [8]. We present the case of a 23-year-old woman diagnosed with TS who was admitted due to hematuria and fever.

## Case presentation

We present the case of a 23-year-old woman with a past medical history of TS, diagnosed at six months of age, which had manifested as CNS and renal angiomyolipomas. In the past, her renal involvement has caused macroscopic hematuria resulting in transfusion support and the need for selective arterial embolization.

On this occasion, she consulted again reporting hematuria. She was admitted to a high-complexity hospital where she was in fair general condition and was found to have a blood pressure of 100/70 mmHg, a heart rate of 110 beats per minute, and an oxygen saturation of 96%.

On physical examination, a systolic ejection murmur in the aortic area was detected. Given the clinical suspicion of a new angiolipoma with hematuria, contrast-enhanced abdominal tomography was performed and bilateral renal angiomyolipomas were documented, causing dilatation of the collecting cavities.

**Figure 1:**
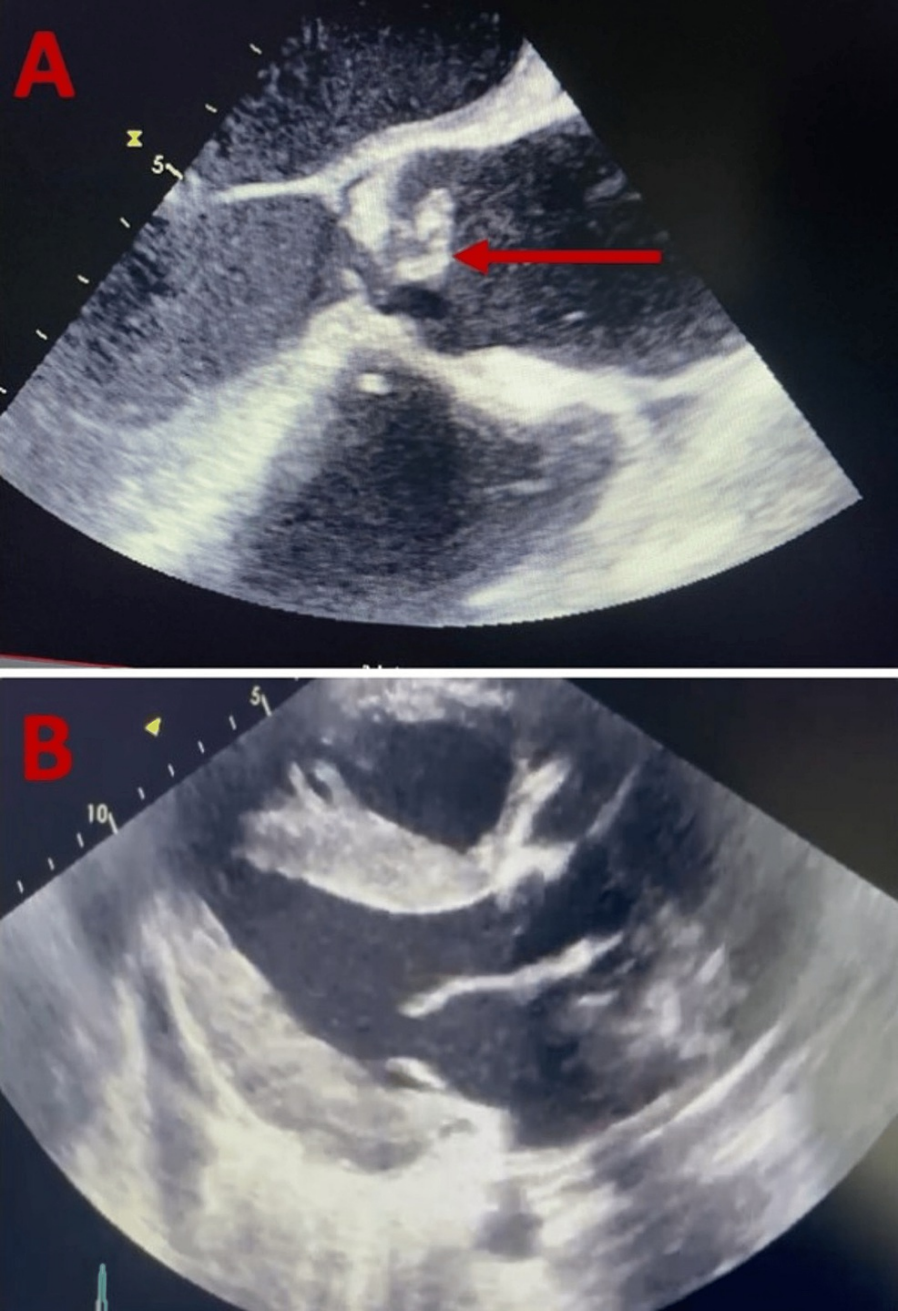
Diagnostic imaging (echocardiography) (A) Parasternal short-axis view with the arrow pointing to a mass attached to the aortic valve (red arrow).​ (B) Postoperative short-axis view showing the absence of the aortic valve mass.

Due to the presence of objective fever, blood cultures were taken where it was possible to isolate *Streptococcus sanguis* in all three cultures drawn, with persistence in positivity despite antibiotic treatment, and a transesophageal echocardiogram (TE) was ordered, taking into account the high risk of infective endocarditis of *S. sanguis*. The TE described a left ventricle with concentric hypertrophy, preserved global contractility, left ventricle ejection fraction (LVEF) of 59%, global longitudinal strain of -15.2%, preserved diastolic function, a tri-leaflet aortic valve with thickening and infiltration of the left coronary leaflet by an echo-dense image of 1 cm^2^ without independent movement of the leaflet (which in the patient's context may correspond to valvular tumor compromise associated with TS or infectious compromise due to endocarditis), with restriction of its opening, but with a valve area of 1.3 cm2 by planimetry, with the presence of a discrete subaortic membrane that causes severe subvalvular stenosis (CW 613 cm/sec, maximum gradient of 150 mmHg, mean gradient of 100 mmHg) with moderate to severe regurgitation (half-pressure time 201 ms) (Figures 1A, 1B).

With the previous findings, three Duke criteria were met for possible infective endocarditis, associated with a double aortic valve lesion with a subvalvular membrane causing considerable gradients. The cardiovascular surgery group considered that the patient met the European Cardiology Association (ESC) criteria for surgical management (persistence of positive blood cultures, locally uncontrolled infection, vegetations > 10 mm associated with severe valvular stenosis or insufficiency) [9]. Studies were expanded at the CNS level by means of magnetic resonance imaging, where angiomyolipoma-type lesions were documented, with a substantial risk of bleeding, for which reason a multidisciplinary group decided to replace a valve with a bioprosthetic due to the risk of bleeding with indefinite oral anticoagulation.

**Figure 2:**
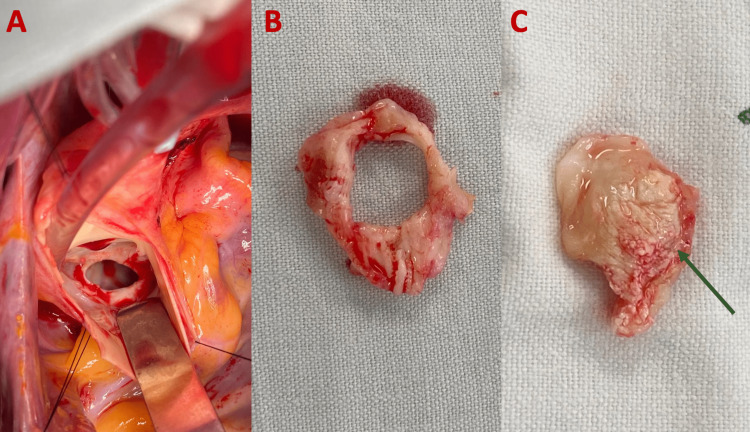
Intraoperative images (A) Aortic native valve during surgery. (B) Resected aortic valve ring. (C) Resected tumor of the aortic valve (green arrow).

Together, a single surgical procedure was carried out for aortic valve replacement with bioprosthesis and resection of the aortic subvalvular membrane, with right radical laparoscopic nephrectomy (Figures 2A-2C).

Histological findings showed fibroconnective tissue with inflammatory lymphoplasmacytic infiltrate, multinucleated giant cells, and extensive areas of necrosis with fibrin, abundant polymorphonuclear neutrophils that form microabscesses, associated with a neoplastic lesion made up of multiple spindle cells with an epithelioid appearance and a clear and eosinophilic cytoplasm, accompanied by vessels and smooth muscle, findings consistent with an epithelioid angiomyolipoma-type neoplastic lesion.

The patient was subsequently discharged with warfarin formulation as an anticoagulant. At a three-month follow-up, she had an adequate post-surgical evolution and denied recurrence of the bleeding. In a multidisciplinary meeting, due to her considerable improvement, her anticoagulation was discontinued.

## Discussion

There is a wide range of cardiac tumors that are classified as benign and malignant. In frequency, the number of secondary tumors is greater than that of the primary ones. When clinicians do encounter a primary cardiac tumor, 90% are benign; but despite this, they can lead to hemodynamic complications of a mechanical type or arrhythmias, depending on their location and size in the heart [10,11].

During childhood, the most common tumors are rhabdomyomas (45%), fibromas, teratomas, and myxomas (15% each) [10]. During adulthood, the most common tumors are myxomas (45%), lipomas (20%), and papillary fibroelastomas (15%) [10]. Although the final diagnosis will be established by histopathology, an imaging study is necessary to evaluate the size, location, relationship with cardiac structures, and hemodynamic parameters. Among the different diagnostic methods, we found echocardiography (in its transthoracic and transesophageal versions), cardiac magnetic resonance imaging (CMRI), cardiac computed tomography (CT), and positron emission tomography (PET/CT) [10,11]. An additional benefit provided by CMRI is to establish the presence of infiltration of surrounding tissues. PET/CT will be useful to assess the metabolic activity of the lesion to help discern whether it is a malignant lesion. In addition to what has been mentioned, some of these methods allow the visualization of thoracic structures, being useful for surgical planning [10,11].

As previously mentioned, the presence of cardiac tumors in the context of TS is a well-known scenario. However, this is common during childhood. The literature mentions the existence of factors that favor disease penetrance into adulthood, associating mild phenotypes, mosaicism, and the effect of disease-modifying gene polymorphisms [8]. The vast majority of patients have mutations in the TSC1 gene, but no mutation is also common in some patients, up to 15% [8].

## Conclusions

TS is a rare genetic disorder that causes the development of benign tumors in various organs, including but not limited to the kidney, CNS, and cardiovascular system. It can lead to symptoms such as seizures, developmental delays, and skin abnormalities. We presented the case of a woman with a history of TS, who was found to have cardiac compromise due to a valvular rhabdomyoma that required surgical intervention. In cases like this, when clinical suspicion arises, in patients with a known history of TS, and due to the risk of heart failure from delayed diagnosis and the asymptomatic nature of these tumors, echocardiography is recommended at any age, even in adolescence or adulthood. A prompt diagnosis and treatment, when feasible, can help prevent further irreversible complications and improve the quality of life for these patients.

## References

[1] Portocarrero LK, Quental KN, Samorano LP, Oliveira ZN, Rivitti-Machado MC (2018). Tuberous sclerosis complex: review based on new diagnostic criteria. An Bras Dermatol.

[2] Randle SC (2017). Tuberous sclerosis complex: a review. Pediatr Ann.

[3] Naderi N, Timofte I, McCurdy MT, Reed RM (2015). Tuberous sclerosis complex: multisystem hamartomas. BMJ Case Rep.

[4] Sandoval-Tress C, Martínez-Baumbach EB, Rodríguez-Mora EA, López-Terrazas JH (2009). Valvular and subvalvular aortic stenosis. Unusual expression of tuberous sclerosis (Article in Spanish). An Pediatr (Barc).

[5] Quek SC, Yip W, Quek ST, Chang SK, Wong ML, Low PS (1998). Cardiac manifestations in tuberous sclerosis: a 10-year review. J Paediatr Child Health.

[6] Devlin LA, Shepherd CH, Crawford H, Morrison PJ (2006). Tuberous sclerosis complex: clinical features, diagnosis, and prevalence within Northern Ireland. Dev Med Child Neurol.

[7] Staley BA, Vail EA, Thiele EA (2011). Tuberous sclerosis complex: diagnostic challenges, presenting symptoms, and commonly missed signs. Pediatrics.

[8] Seibert D, Hong C hui, Takeuchi F, Olsen C, Hathaway O (2011). Annals of internal medicine recognition of tuberous sclerosis in adult women: delayed presentation with life-threatening consequences. Ann Intern Med.

[9] Habib G, Lancellotti P, Antunes MJ (2015). 2015 ESC Guidelines for the Management of Infective Endocarditis: The Task Force for the Management of Infective Endocarditis of the European Society of Cardiology (ESC). Endorsed by: European Association for Cardio-Thoracic Surgery (EACTS), the European Association of Nuclear Medicine (EANM). Eur Heart J.

[10] Tyebally S, Chen D, Bhattacharyya S (2020). Cardiac Tumors: JACC CardioOncology State-of-the-Art Review. JACC CardioOncol.

[11] Poterucha TJ, Kochav J, O'Connor DS, Rosner GF (2019). Cardiac tumors: clinical presentation, diagnosis, and management. Curr Treat Options Oncol.

